# MRI findings in a dog with calvarial squamous cell carcinoma

**DOI:** 10.1111/jsap.70102

**Published:** 2026-02-13

**Authors:** P. Sebestyén, U. Teubenbacher, G. Rosato, I. Lautenschläger, L. Golini

**Affiliations:** ^1^ Section of Neurology, Department of Small Animals, Vetsuisse Faculty University of Zurich , Zurich Switzerland; ^2^ Clinic for Diagnostic Imaging, Department of Clinical Diagnostics and Services, Vetsuisse Faculty University of Zurich Zurich Switzerland; ^3^ Institute of Veterinary Pathology, Vetsuisse Faculty University of Zurich Zurich Switzerland

A 9‐year‐old spayed female Jack Russell terrier was presented with a history of apathy, anorexia and progressive cranial deformation over several weeks. There was no history of trauma. Clinical examination revealed bilateral exophthalmos and a painful, crepitant, frontotemporal mass. Neurological examination showed mild obtundation and reduced menace response, indicating forebrain involvement. Bloodwork was unremarkable. MRI (Ingenia 3.0T, Philips AG, Zurich, Switzerland) revealed an extensive, polyostotic calvarial lesion with permeative osteolysis and osteoproliferation. The lesion caused severe, bilateral destructive sinorhinopathy, retrobulbar extension with exophthalmos, multifocal masticatory muscle infiltration and dural/meningeal enhancement. On T2‐weighted fat‐suppressed (T2wSPIR) sequences, it was predominantly hyperintense relative to the masticatory musculature, with multifocal T2‐weighted hyperintense areas (Fig 1A). The lesion also displayed a thick, irregular, enhancing rim and multifocal to confluent regions of marked contrast enhancement on T1‐weighted sequences after gadolinium‐based contrast medium injection (post‐contrast T1w, Fig 1B). Differential diagnoses included neoplasia (*e.g*. carcinoma, multilobular osteochondrosarcoma or soft tissue sarcoma), while inflammatory/infectious aetiologies (*e.g*. fungal osteomyelitis) were considered less likely. *Post mortem* examination revealed cranial deformities, exophthalmos and neoplastic infiltration of skull bones, meninges and temporal region, obstructing the caudal nasal cavity (Fig 1C). Histopathology confirmed a highly cellular, infiltrative squamous cell carcinoma (SCC) with high mitotic activity and vascular emboli (Fig 1D). SCC is a malignant epithelial tumour, and diffuse calvarial infiltration with extensive osteolysis and concurrent osteoproliferation, as in this case, is uncommon. This report highlights that SCC should be considered among differentials for polyostotic, osteolytic and osteoproliferative calvarial lesions with similar MRI features in dogs.

**FIG. 1:**
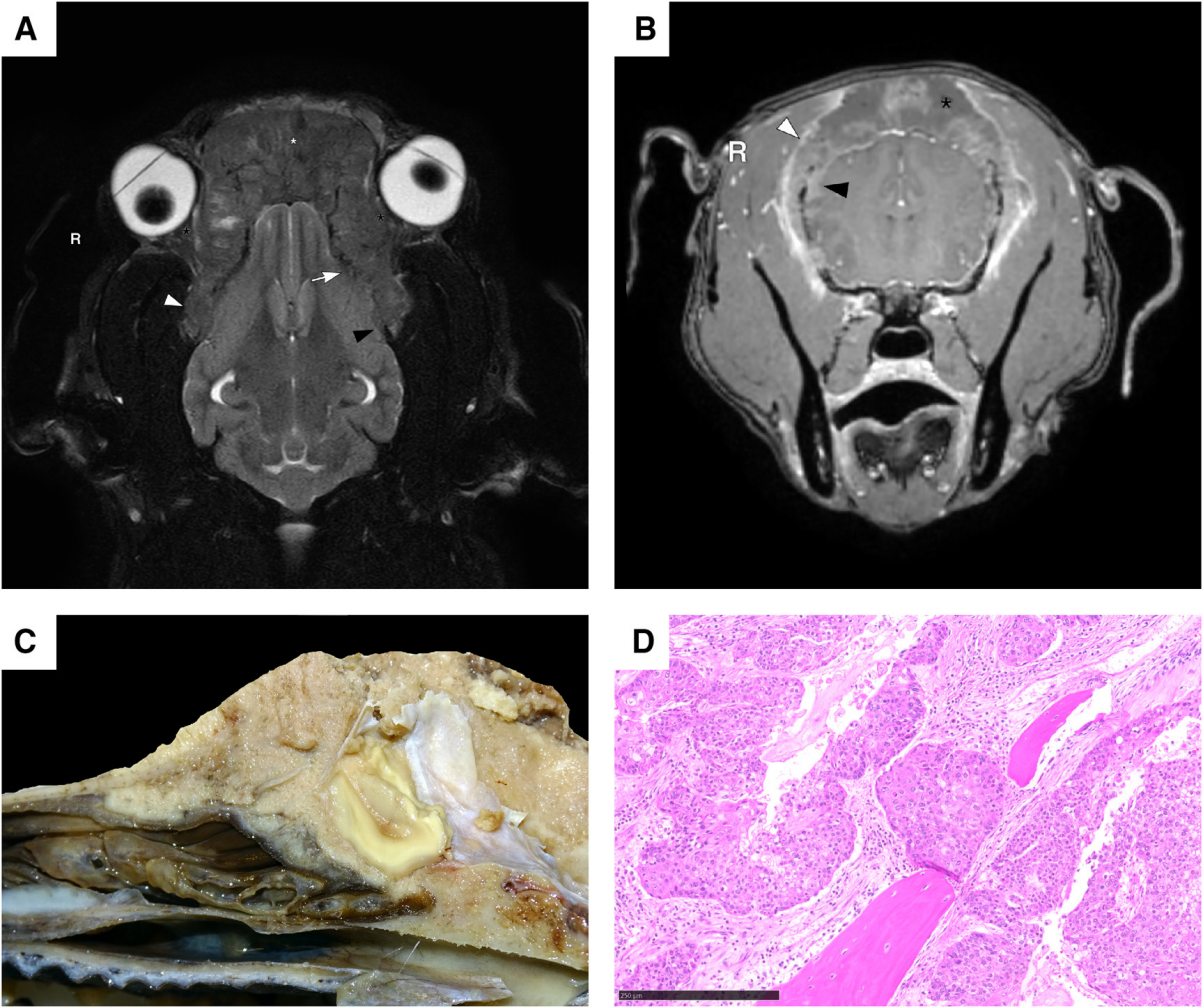
(A) Dorsal T2wSPIR: severe sinorhinopathy (white star), masticatory muscle infiltration (white arrowhead), calvarial lysis (white arrow), meningeal infiltration (black arrowhead), and bilateral exophthalmos (black stars). (B) Transverse post‐contrast T1w: mass replacing the calvarium (black star), enhancing rim (white arrowhead), and meningeal infiltration (black arrowhead). (C) Post‐mortem median skull section. (D) Histopathology (H&E, scale bar = 250 µm)

## Author contributions

**P. Sebestyén:** Conceptualization; writing – original draft; writing – review and editing. **Ursula Teubenbacher:** Conceptualization; writing – original draft; writing – review and editing; **Guiliana Rosato:** Conceptualization; writing – original draft; writing – review and editing; **Ines Lautenschläger:** Conceptualization; writing – original draft; writing – review and editing; **Lorenzo Golini:** Conceptualization; writing – original draft; writing – review and editing;

